# Heterotopic Ossification: A Late Complication From a Chemical Burn

**Published:** 2018-01-15

**Authors:** Eric Clayman, Bahar Abbassi, Anthony W. Watt, Wyatt G. Payne

**Affiliations:** ^a^University of South Florida Morsani College of Medicine, Tampa; ^b^Division of Plastic Surgery, Department of Surgery, University of South Florida Morsani College of Medicine, Tampa; ^c^C. W. Bill Young Bay Pines VA Medical Center, Bay Pines, Fla

**Keywords:** heterotopic ossification, burn, burn complications, bone tissue, burn reconstruction

**Figure 1:**
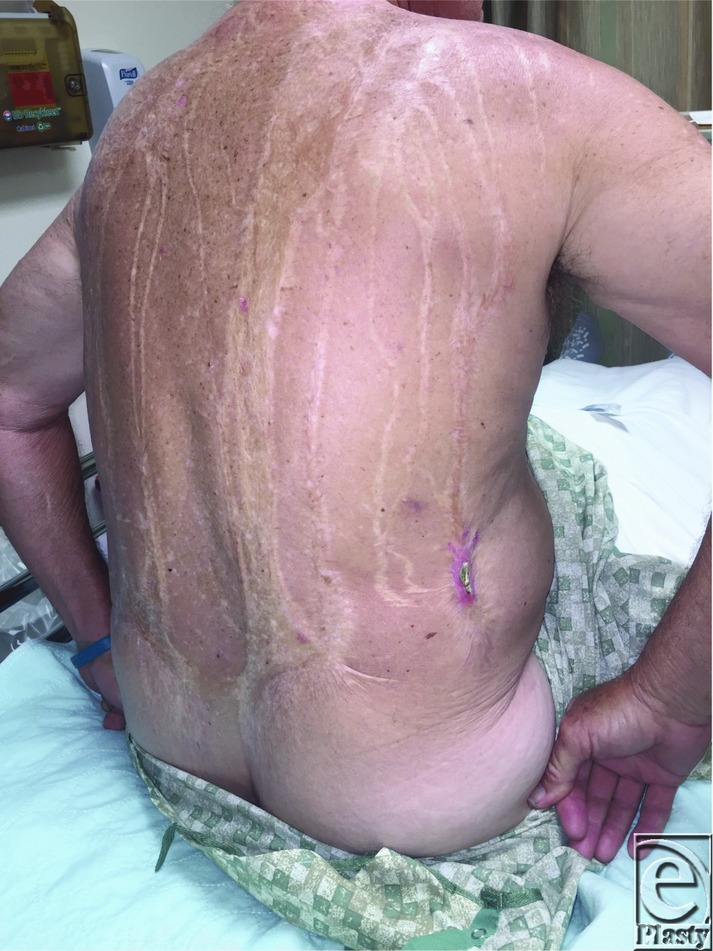


## DESCRIPTION

A 70-year-old man with a history of napalm burn to the posterior torso that he sustained while serving in the military during the Vietnam War presented with a firm mass on his right flank. Three years prior to this presentation, he developed a similar lesion on his left flank that was excised, with pathology confirming a diagnosis of heterotopic ossification (HO).

## QUESTIONS

1. What is heterotopic ossification?
2. What causes heterotopic ossification?
3. What is the standard method of diagnosis?
4. What is the preferred management of heterotopic ossification?

## DISCUSSION

Heterotopic ossification is a relatively uncommon and debilitating complication associated with burns, spinal cord/neurologic injury, musculoskeletal trauma, and orthopedic surgery.^1^ It is defined as ectopic production of mature bone in nonskeletal tissue.^2^ Heterotopic ossification can occur anywhere on the body but has commonly been described as occurring on the upper extremities and overlying the hips. Cutaneous HO is largely asymptomatic; however, joint involvement often presents with pain and limitations in range of motion secondary to ankylosis. Interestingly, HO that occurs following burn injuries is not always limited to the site of cutaneous injury.^3^

While the exact cause of HO is poorly defined, several research studies highlight the inflammatory response as well as infectious causes as precipitators for the disease. Studies found that the severity and incidence of HO is directly correlated to the degree of injury and inflammatory response of the inciting event.^1^ Early inflammatory markers, such as interleukin (IL)-3, IL-12, and IL-13, have been found to be associated with the development of HO when studied in combat-related blast injuries.^4^ These cytokines play important roles in lymphocyte differentiation as well as bone homeostasis through inhibiting osteoclastic activity by upregulating osteoprotegerin.^1^^,^^5^ As such, current research is investigating the role of the adaptive immune response in the development of HO.

Much attention has been paid in predicting the development of HO in patients with spinal cord injury and in those with combat-related injuries. Ultrasonography has been shown to be a reliable and highly sensitive screening modality for the diagnosis of HO in this patient population, with a sensitivity of 88.9%.^6^ The most sensitive imaging modality for early detection and for assessing the maturity of HO is a 3-phase technetium-99m (99mTc) methylene diphosphonate bone scan.^2^ The diagnosis of HO can be confirmed with magnetic resonance imaging or computed tomography.^7^

There are various management options that may be utilized in preventing and/or treating HO. Physical therapy interventions are aimed at maintaining joint mobility but are controversial in HO prevention and treatment, as various studies have shown that passive stretching has been associated with progression of ossification, eventually leading to complete ankylosis. However, other studies have found that daily aggressive stretch exercises have caused significant improvement in joint motion and can eliminate the need for further surgery.^8^ In addition to physical therapy interventions, indomethacin is frequently used in the prophylaxis and early treatment of HO due to its ability to prevent the inflammatory response that has been associated with HO development. Radiation therapy has been shown to be effective in the prophylaxis and prevention of progression of HO as a result of inhibition of mesenchymal cell differentiation. Pulse low-intensity electromagnetic field therapy, which utilizes magnetic fields to increase oxygen levels and decrease toxic by-products of inflammation by increasing local blood flow, has been shown to be effective in preventing the development of HO. Bisphosphonates have been shown to halt the progression of HO due to their role in preventing bone mineralization. Surgical excision should be delayed 12 to 18 months after development of HO until radiographic evidence of HO maturation. In addition, surgical excision should be supplemented with bisphosphonate therapy to prevent secondary HO formation.^9^

Our patient had a history of napalm burn to the posterior torso sustained in combat with the eventual development of HO on his left flank, which was excised and subsequently developed HO on the right flank 3 years later. Of note, he did not receive supplemental treatment with the aforementioned nonsurgical therapies, which may have prevented his second episode of HO.
